# Acute kidney injury in patients undergoing cardiac transplantation: a cohort study on incidence and risk factors in the first three years of service implementation

**DOI:** 10.1590/2175-8239-JBN-2025-0135en

**Published:** 2026-03-09

**Authors:** Mariana Moura Ferreira, Marcello Laneza Felicio, Flávio de Souza Brito, Leonardo Rufino Garcia, Daniela Ponce

**Affiliations:** 1 Universidade Estadual Paulista “Júlio de Mesquita Filho” (UNESP), Faculdade de Medicina de Botucatu, Curso de Medicina, Botucatu, SP, Brazil.; 2 Universidade Estadual Paulista “Júlio de Mesquita Filho” (UNESP), Faculdade de Medicina de Botucatu, Serviço de Cirurgia Cardiovascular, Botucatu, SP, Brazil.; 3 Hospital Estadual de Bauru, Serviço de Cirurgia Cardíaca, Bauru, SP, Brazil.; 4 Universidade Estadual Paulista “Júlio de Mesquita Filho” (UNESP), Faculdade de Medicina de Botucatu, Programa de Transplante Cardíaco, Botucatu, SP, Brazil.; 5 Centro de Pesquisa Clínica Indacor, Indaiatuba, SP, Brazil.; 6 Faculdade de Medicina UniMAX, Curso de Medicina, Indaiatuba, SP, Brazil.; 7 Universidade Estadual Paulista “Júlio de Mesquita Filho” (UNESP), Faculdade de Medicina de Botucatu, Unidade de Diálise do Hospital das Clínicas, Botucatu, SP, Brazil.

**Keywords:** Acute Kidney Injury, Heart Transplantation, Cardiopulmonary Bypass, Extracorporeal Circulation, Mortality, Risk factors

## Abstract

**Introduction:**

Acute kidney injury (AKI) is a common complication in the postoperative period of heart transplantation and is associated with unfavorable patient outcomes.

**Objective:**

To analyze the incidence of AKI in patients undergoing heart transplantation and to identify preoperative, intraoperative, and postoperative risk factors associated with its development.

**Methods:**

This was a single-center retrospective cohort study including patients who underwent heart transplantation during the first three years of the program at a tertiary hospital in the State of São Paulo, from January 2020 to January 2023. Patients with chronic kidney disease (CKD) stages 4 or 5 and prior kidney transplantation were excluded.

**Results:**

The incidence of AKI was 48%. Logistic regression analysis demonstrated an association between AKI and the following factors: pre-existing CKD (OR = 3.155; 95% CI 1.343–6.340; p = 0.031), cold ischemia time (OR = 1.956; 95% CI 1.126–3.053; p = 0.042), and higher doses of norepinephrine in the first postoperative day (OR = 5.211; 95% CI 2.696–8.987; p = 0.028). There was no significant difference in mortality between patients who developed AKI and those who did not (58.3% vs. 38.5%; p = 0.09).

**Conclusion:**

The incidence of AKI was high (48%) in this population. The main risk factors for its development were pre-existing CKD, prolonged cold ischemia time, and higher doses of norepinephrine in the first postoperative day.

## Introduction

Acute kidney injury (AKI) is a frequent complication in patients undergoing heart transplant surgery. It is associated with increased morbidity and mortality, as well as prolonged hospitalization and higher healthcare costs^1,2,3,4^. According to a systematic review published by Thongprayoon et al. in 2019, the incidence of AKI after heart transplantation was 62%^4^. Therefore, its prevention and understanding of its pathophysiology are topics of interest.

The pathophysiology of AKI is not unique and is associated with its own etiology. The injury may originate from renal hypoperfusion, exposure to nephrotoxic agents, sepsis, and immunosuppression, among other causes^1,2,3,4^.

Among the identified risk factors are the preoperative ones, including chronic kidney disease (CKD), diabetes mellitus (DM), and advanced age^5,6,7^. Perioperative and postoperative (PO) risk factors include increased cardiopulmonary bypass (CPB) and surgery duration, prolonged cold ischemia time (CIT), supratherapeutic concentrations of calcineurin inhibitors, PO bleeding, the need for right ventricular assist devices, the duration of mechanical ventilation (MV), and the need for extracorporeal membrane oxygenation (ECMO) after heart transplantation^8,9,10^.

Despite the remarkable advances in medicine, specifically in the fields of heart transplantation and nephrology, data on the incidence and risk factors associated with AKI in heart transplant patients remain scarce, particularly in developing countries.

Identifying these risk factors enables targeted interventions to mitigate them. Thus, this study aims to identify risk factors that may increase the likelihood of heart transplant recipients developing AKI within the first PO week, whether they are pre-existing, present at the time of surgery, or post-surgical.

## Methods

A retrospective observational cohort study was conducted involving adult patients who underwent heart transplantation at the *Hospital das Clínicas da Faculdade de Medicina de Botucatu* (HCFMB) between January 2020 and January 2023.

Patients with stages 4 and 5 CKD (creatinine clearance below 30 ml/min), kidney transplant recipients, and those who had AKI prior to heart transplantation were excluded from the study.

The study was reviewed and approved by the Research Ethics Committee of the *Faculdade de Medicina de Botucatu* (FMB) under opinion No. 5,977,493, and was only initiated after obtaining ethical clearance.

Preoperative data such as age, weight, height (with calculation of body mass index – BMI), presence of comorbidities such as diabetes, hypertension, and obesity, baseline creatinine, prognostic indices, etiology of heart failure, and echocardiogram findings (ejection fraction [EF], septal and posterior wall thickness, pulmonary hypertension) were collected. Intraoperative data were also collected, such as surgery duration, cold ischemia time, CPB duration, and intraoperative complications, including hemodynamic instability, need for vasoactive drugs (VAD), and blood transfusion. In addition, the following clinical and laboratory variables were assessed daily, both preoperatively and during the first three days of the PO: diuresis, need for mechanical ventilation and fraction of inspired oxygen (FiO2), use of vasoactive drugs, temperature, hemoglobin, albumin, C-reactive protein, and white blood cell count.

The definition and classification of AKI used in this study followed the 2012 Kidney Disease Improving Global Outcomes (KDIGO) guidelines, based on either an increase in creatinine (≥0.3 mg/dL in 48 hours or ≥1.5 in 7 days) or a decrease in urine output (<0.5 mL/kg/h for more than 6 hours)^11^.

Immunosuppressants were prescribed according to an institutional protocol based on the 2018 Brazilian Guidelines for Cardiac Transplantation^12^ and consisted of triple therapy (prednisone, mycophenolate mofetil, and tacrolimus). In cases where Chagas disease was the underlying condition, mycophenolate was replaced with azathioprine.

Based on the study protocol, data were entered into an electronic spreadsheet, checking for possible typing errors, and analyzed using IBM SPSS for Windows, version 17.0. Initially, a descriptive analysis of the patients followed up during the period was performed, with measures of central tendency and dispersion calculated for continuous variables, and frequency for categorical variables. Subsequently, the occurrence of AKI was established as the dependent variable.

The Chi-square test was used to analyze categorical variables; the t-test was applied to compare continuous and parametric variables; and the Mann-Whitney test was used for nonparametric variables. Multivariate analysis was performed using logistic regression, including in the model all variables that showed an association with the outcome (p ≤ 0.05) in the univariate analyses, after excluding those considered collinear, as identified by the Kolmogorov-Smirnov test. The backward selection method was applied iteratively to remove non-significant variables. Variables that, when removed, resulted in a change greater than 10% in the parameter were reintroduced into the model to control for potential confounding factors. A significance level of 5% was adopted for all tests performed.

## Results

**Figure 1.**
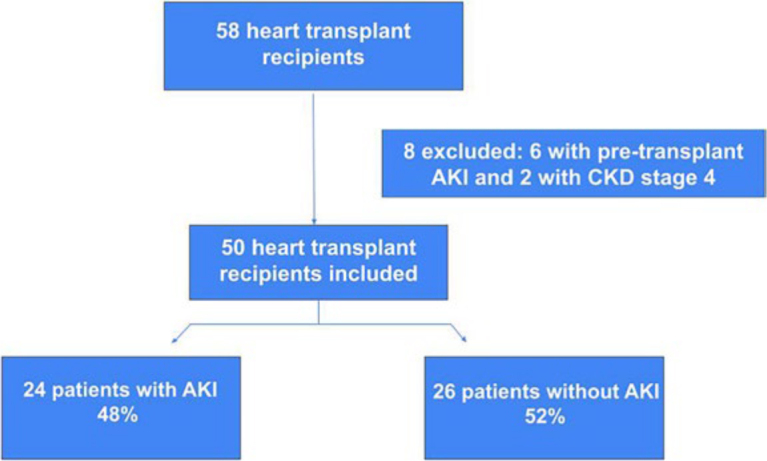
Flowchart of patient selection.

During the study period, 58 patients underwent heart transplant surgery at the *Hospital das Clínicas de Botucatu*. Of these, eight were excluded due to the presence of preoperative AKI or stage 4 CKD, as shown in Figure 1. Most patients were male (76%) and Caucasian (84%), with a mean age of 48 ± 12 years. The main etiologies of heart failure were ischemic (32%) and Chagasic (28%). The most frequent comorbidities were hypertension (32%), type 2 diabetes mellitus (16%), and CKD (18%). The patients’ baseline creatinine was 0.89 ± 0.32 mg/dL, EF was 23.9 ± 10%, left ventricular (LV) wall thickness was 8.9 ± 1.9, and pulmonary artery pressure was 49.5 ± 15.5 mmHg. The vast majority of patients had reduced EF (90%) and pulmonary hypertension (85%), as shown in Table 1. Preoperative hemoglobin and albumin concentrations were 11.96 ± 2.13 and 3.70 ± 0.84 g/dL, respectively. Most patients were receiving positive inotropic therapy (80%), and 12 were using an intra-aortic balloon pump (IABP) (24%) preoperatively.

Abbreviations – AKI: acute kidney injury; CKD: chronic kidney disease.

**Table 1:** Preoperative clinical and laboratory characteristics of patients according to the development of aki during hospitalization

Variables	Overall (N = 50)	AKI (N = 24)	No AKI (N = 26)	P-value
Male (%)	38 (76)	19 (79.2)	19 (73.1)	0.614
Age (Years)	48 ± 12	44 ± 10	51 ± 12	0.02
Race (%)				0.364
White	42 (84%)	22 (91.7%)	20 (76.9%)	
Black	4 (8%)	1 (4.2%)	3 (11.5%)	
Comorbidities (%)				
Obesity	4 (8)	2 (8.3)	2 (7.6)	0.709
CKD	9 (18%)	8 (33.3%)	1 (3.8%)	0.007
DM	8 (16%)	4 (16.7%)	4 (15.4%)	0.902
Hypertension	16 (32%)	8 (33.3%)	8 (30.8%)	0.846
Thyroid disease	7 (14%)	5 (20.8%)	2 (7.7%)	0.274
Former smoker	13 (26%)	5 (20.8%)	8 (30.8%)	0.422
Former alcohol use	7 (14%)	3 (12.5%)	4 (15.4%)	0.587
Other (%)^*^	19 (38%)	10 (41.7%)	9 (34.6%)	0.575
Type of HF (%)				0.605
Ischemic	13 (26%)	6 (25%)	7 (26.9%)	
Valvular	4 (8%)	2 (8.3%)	2 (7.7%)	
Chagas disease	14 (28%)	9 (37.5%)	5 (19.2%)	
Other^**^	16 (32%)	6 (25%)	8 (29.7%)	
Echocardiogram data				
EF (%)	23.9 ± 10	26.1 ± 11.2	21.4 ± 8.4	0.03
LV thickness (mm)	8.9 ± 1.9	9.5 ± 2.1	8.5 ± 1.5	0.249
Pulmonary artery pressure (mmHg)	49.5 ± 15.5	51.5 ± 16.5	48.8 ± 13.9	0.263
Preoperative hemoglobin (g/dL)	11.96 ± 2.13	11.64 ± 2.31	12.24 ± 1.95	0.336
Preoperative albumin (g/dL)	3.70 ± 0.84	3.43 ± 0.85	3.90 ± 0.80	0.086
Preoperative CPR (mg/dL)	3.93 ± 5.82	4.97 ± 7.12	2.89 ± 4.04	0.251
Preoperative leukocytes (n°/mm^3^)	8.51 ± 3.46	8.86 ± 3.94	8.20 ± 3.03	0.514
Use of inotropic agents (%)	40 (80)	20 (83)	20 (76.9)	0.621
Use of IAB (%)	12 (24)	9 (37.5)	3 (11.4)	0.108

Abbreviations – CKD, chronic kidney disease; CRP, C-reactive protein; DM, diabetes mellitus; EF, ejection fraction; HF, heart failure; IAB, intra-aortic balloon; LV, left ventricle.

Notes – ^*^Chronic arterial disease, Sjögren's syndrome, depressive disorder, illicit drug user, trigeminal neuralgia, etc.

**Viral myocarditis, dilated cardiomyopathy (to be clarified), ischemic + valvular, ischemic + chagas.

The incidence of AKI was 48%. The mean time to AKI development was 2.79 ± 1.06 days, ranging from 1 to 5 days. There was a predominance of KDIGO 3 AKI (10 patients – 41.7%), followed by KDIGO 2 AKI (7 patients – 29.1%), and CKD with superimposed AKI (6 patients – 25%). Artificial kidney support (AKS) was indicated in 19 patients (38%). The predominant AKS modality was prolonged hemodialysis (9 patients – 47.4%), followed by continuous venovenous therapy (6 patients – 31.6%), conventional intermittent hemodialysis (2 patients – 10.5%), and peritoneal dialysis (2 patients – 10.5%).

The preoperative factors identified as associated with AKI were age (44 ± 10 vs. 51 ± 12 years; p = 0.02), EF (26.3 ± 11.2 vs. 21.4 ± 8.6; p = 0.03), and the presence of CKD (3.8 vs. 33%; p = 0.007). There were no differences between patients with and without AKI regarding sex, comorbidities, pulmonary artery pressure, or the use of inotropic agents or devices in the preoperative period.

During the intraoperative period, the vast majority of patients required vasoactive drugs (96%) and blood transfusions (96%). The total CPB time was 264.9 ± 59 min, cold ischemia time was 169.97 ± 75.49 min, and warm ischemia time was 117.71 ± 41.31 min. The groups with and without AKI were similar regarding the need for and duration of CPB, use of vasoactive drugs, need for and amount of blood transfusion, and warm ischemia time. However, they differed with regard to cold ischemia time, which was longer in patients who developed AKI (196.83 ± 66.10 min vs. 141.53 ± 76.22; p = 0.028), as shown in Table 2.

**Table 2:** Analysis of intraoperative characteristics of patients who developed aki during hospitalization

Variables	Overall (N = 50)	AKI (N = 24)	No AKI (N = 26)	P-value
Use of CPB (%)	47 (94%)	24 (100%)	23 (88.5%)	0.229
Use of vasoactive drugs during surgery (%)				
Norepinephrine	34 (68%)	18 (75%)	16 (61.5%)	0.440
Dobutamine	48 (96%)	24 (100%)	24 (92.3%)	0.382
Milrinone	40 (80%)	20 (83.3%)	20 (76.9%)	0.597
Epinephrine	26 (52%)	14 (58.3%)	12 (46.2%)	0.480
Surgical complications (%)				0.620
Significant bleeding	4 (8%)	3 (12.5%)	1 (3.8%)	
Vasoplegic shock	2 (4%)	1 (4.2%)	1 (3.8%)	
Other	6 (12%)	4 (16.7%)	2 (7.7%)	
Total CPB time (minutes)	264.89 ± 59.97	274.13 ± 68.05	254.82 ± 49.32	0.280
Cold ischemia time (minutes)	169.97 ± 75.49	196.83 ± 66.10	141.53 ± 76.22	0.028
Warm ischemia time (minutes)	117.71 ± 41.31	125.12 ± 26.50	110.29 ± 51.97	0.303
Use of blood products – red blood cells (units)	4.09 ± 2.79	4.43 ± 2.42	3.74 ± 3.13	0.405
Use of blood products – fresh frozen plasma (units)	2.59 ± 1.99	2.61 ± 1.87	2.57 ± 2.15	0.942
Use of blood products – cryoprecipitate (units)	2.87 ± 3.27	2.74 ± 3.29	3.00 ± 3.31	0.790
Use of blood products – plateletpheresis (units)	0.99 ± 0.62	1.09 ± 0.57	0.89 ± 0.67	0.296

Abbreviations – CPB, cardiopulmonary by-pass.

The factors associated with AKI on the first PO day were the need for VAD (95.8% vs. 65.4%; p = 0.034), norepinephrine dose (0.57 ± 0.23 vs. 0.18 ± 0.06 mcg/kg/min; p = 0.041), higher CRP values (16.78 ± 9.23 vs. 10.77 ± 6.72 mg/dl; p = 0.026), and greater need for mechanical ventilation (50 vs. 15.4%; p = 0.014). There were no differences between patients with and without AKI regarding FiO2, maximum temperature, hemoglobin, albumin, and leukocyte values, as shown in Table 3.

**Table 3:** Variables analyzed on the 1st and 3rd postoperative days regarding the development of aki during hospitalization

Variables	Overall (N = 50)	AKI (N = 24)	No AKI (N = 26)	P-value
FiO2 on 1st PO day (%)	49.68 ± 21.63	54.16 ± 23.33	36.25 ± 4.78	0.158
Use of vasoactive drugs (%)	40 (80)	23 (95.8)	17 (65.4)	0.034
Vasoactive drugs on 1st PO day – norepinephrine (mcg/kg/min)	0.36 ± 0.62	0.57 ± 0.53	0.18 ± 0.66	0.041
Vasoactive drugs on 1st PO day – dobutamine (mcg/kg/min)	9.47 ± 3.22	9.65 ± 3.71	9.33 ± 2.81	0.749
Vasoactive drugs on 1st PO day – milrinone (mcg/kg/min)	0.49 ± 0.29	0.52 ± 0.33	0.46 ± 0.26	0.564
Vasoactive drugs on 1st PO day – epinephrine (mcg/kg/min)	0.24 ± 0.42	0.42 ± 0.55	0.08 ± 0.16	0.008
Maximum temperature on 1st PO day (°C)	37.44 ± 0.92	37.67 ± 1.06	37.24 ± 0.73	0.132
Hemoglobin on 1st PO day (g/dL)	9.12 ± 1.23	9.52 ± 1.09	8.94 ± 1.30	0.127
Albumin on 1st PO day (g/dL)	2.68 ± 0.43	2.67 ± 0.48	2.70 ± 0.38	0.826
CRP on 1st PO day (mg/dL)	13.30 ± 8.32	16.78 ± 9.23	10.77 ± 6.72	0.026
Leukocytes on 1st PO day (n°/mm^3^)	16.72 ± 7.39	18.05 ± 8.76	15.56 ± 5.92	0.277
Mechanical ventilation on 1st PO day (%)	16 (32%)	12 (50%)	4 (15.4%)	0.014
FiO2 on 3rd PO day (%)	37.77 ± 11.48	38.75 ± 11.87	30.00	0.510
Use of vasoactive drugs (%)	32 (64)	18 (75)	14 (53.8)	0.314
Vasoactive drugs on 3rd PO day – norepinephrine (mcg/kg/min)	0.24 ± 0.51	0.56 ± 0.66	0.00	<0.001
Vasoactive drugs on 3rd PO day – dobutamine (mcg/kg/min)	8.95 ± 3.29	10.35 ± 3.47	7.86 ± 2.76	0.015
Vasoactive drugs on 3rd PO day – milrinone (mcg/kg/min)	0.50 ± 0.29	0.56 ± 0.28	0.46 ± 0.30	0.300
Vasoactive drugs on 3rd PO day – epinephrine (mcg/kg/min)	0.07 ± 0.21	0.15 ± 0.30	0.01 ± 0.04	0.036
Maximum temperature on 3rd PO day (°C)	36.60 ± 0.57	36.46 ± 0.59	36.71 ± 0.55	0.179
Hemoglobin on 3rd PO day (g/dL)	8.77 ± 1.23	9.07 ± 1.68	8.53 ± 0.67	0.170
Albumin on 3rd PO day (g/dL)	2.73 ± 0.37	2.70 ± 0.39	2.77 ± 0.36	0.568
CRP on 3rd PO day (mg/dL)	9.83 ± 933	12.76 ± 11.98	7.92 ± 6.73	0.119
Leukocytes on 3rd PO day (n°/mm^3^)	15.31 ± 7.28	17.66 ± 8.48	13.47 ± 5.71	0.067
Mechanical ventilation on 3rd PO day (%)	8 (16%)	7 (29.2%)	1 (3.8%)	0.011
Use of nephrotoxic drugs on the first 3 postoperative days (aminoglycoside and/or vancomycin)	14 (28%)	8 (33.3%)	6 (23.1%)	0.317

Abbreviations – CRP, C-reactive protein; FiO2, fraction of inspired oxygen; PO, postoperative.

On the third PO day, the need for mechanical ventilation (29.2% vs. 3.8%; p = 0.011) and higher norepinephrine doses remained associated with the incidence of AKI (0.56 ± 0.66 vs. 0; p < 0.001). The other parameters, such as FiO2, other vasoactive drugs used, maximum temperature, hemoglobin, albumin, C-reactive protein, leukometry, and use of nephrotoxic drugs (such as aminoglycosides and vancomycin), were not associated with AKI, as shown in Table 3. All patients included in the study received calcineurin inhibitors in the immunosuppression regimen.

Finally, a multivariate analysis was performed by constructing a logistic regression model using the backward stepwise method. The variables EF, CKD, CIT, and norepinephrine dose on the first PO day were included in the model, all of which were considered clinically relevant and had p < 0.05 in the univariate analysis. The variables age, use of VAD, CRP, and need for MV were excluded, as they were identified as collinear with EF and norepinephrine dose on the first postoperative day.

When performing the logistic regression analysis, presented in Table 4, it was observed that CKD, cold ischemia time, and the dose of norepinephrine on the first PO day were independently associated with the occurrence of AKI, as shown in the table.

**Table 4:** Logistic regression analysis for aki

Variables	OR	CI 95%	p-value
CKD	3.155	1.343–6.340	0.031
Cold ischemia time (minutes)	1.956	1.126–3.053	0.042
EF (%)	1.423	0.962–2.784	0.145
Norepinefrin dose on first postoperative day (0.1 mcg/kg/min)	5.211	2.696–8.987	0.041

Abbreviations – CKD, chronic kidney disease; EF, ejection fraction.

The overall mortality rate among heart transplant recipients was 42%, with no difference between patients with and without AKI (58.3% vs. 38.5%; p = 0.09).

## Discussion

Our results demonstrate that the incidence of AKI in the postoperative period of patients undergoing heart transplantation is high (48%) and occurs, on average, 2.79 ± 1.06 days after surgery, with a predominance of KDIGO 3 AKI (41.7%). The presence of CKD, longer cold ischemia time, and higher doses of norepinephrine on the first postoperative day are associated with the occurrence of AKI in patients undergoing heart transplantation.

According to a systematic review published by Thongprayoon et al. in 2019, the incidence of AKI in the PO period of heart transplantation is high, reaching 62.8%. Therefore, its prevention and recognition of risk factors are necessary, since AKI is associated with increased mortality rates. However, studies conducted in Latin American populations and developing countries remain scarce.

Previous cohort studies have identified several perioperative, intraoperative, and postoperative risk factors for the development of AKI in heart transplant recipients, such as older age, presence of comorbidities (including CKD, diabetes, hypertension, and smoking), prolonged ischemia and CPB time, need for ECMO, supratherapeutic concentrations of calcineurin inhibitors, pulmonary hypertension, duration of mechanical ventilation and use of norepinephrine, in addition to multiple blood transfusions^3,4,5,6,7,8,13,14,15,16,17^.

In accordance with current literature, our results show that age^16,17,18^, presence of CKD^14,15,16,17^, lower EF, longer cold ischemia time^17^, need for VAD and mechanical ventilation on the first and third postoperative days^15,16,17^, as well as higher CRP levels, are associated with the development of AKI. However, among all these variables, only the presence of CKD, cold ischemia time, and higher norepinephrine doses remained significant in our multivariate logistic regression analysis.

CKD is an important factor to be considered, as patients with this condition already have impaired kidney function, which increases their susceptibility to renal stressors during and after surgery. The reduction in renal reserve makes their response insufficient to pressure fluctuations, the potential use of nephrotoxic drugs, and other possible renal insults. For this reason, CKD is considered a risk factor for the occurrence of AKI^14,15,16,17^. Furthermore, CKD may be present in patients with more severe heart disease, i.e., those with type 2 cardiorenal syndrome, acting as an indirect marker of the severity of heart failure itself. In the present study, collinearity was observed among baseline creatinine, EF, and age, which corroborates this possible association. Renal senescence renders renal responses and their autoregulation slower, increasing susceptibility to AKI. In addition, the natural aging of the kidneys may reduce their ability to recover after stressful events, such as heart transplant surgery^13,21,22,23,24,25^.

Previous studies have also identified the use of vasoactive drugs as a predictor of AKI. Such drugs are often required to maintain mean arterial pressure above 65 mmHg during and after surgery^15,16,17^. However, when their use becomes necessary after the immediate postoperative period, this may reflect complications associated with the surgical procedure, such as severe bleeding, or be associated with infectious conditions. In the present study, higher doses of norepinephrine on the first postoperative day were associated with the development of AKI, which likely reflects acquired infectious complications, since there was collinearity among vasopressor dose, plasma CRP concentrations, and the need for mechanical ventilation.

Finally, few studies have demonstrated that cold ischemia time – that is, the period during which the transplanted heart remains without blood circulation and oxygen during the procedure – has an impact on the risk of AKI^17^. The longer this period, the greater the risk of damage to the transplanted heart and to other organs, such as the kidneys.

Graft ischemia time and troponin release are known predictors of initial poor graft function^17^. The shortage of heart donors means that marginal and geographically distant donors must be evaluated and used. The transplant center in the current study is located in a country of continental dimensions, approximately 250 km from the state capital, São Paulo. Prolonged ischemia leads to greater systemic damage resulting from oxidative stress, inflammation, and ischemia itself. Two previous studies have demonstrated the impact of this phenomenon on early postoperative morbidity, including primary graft failure, the need for ECMO, and the occurrence of AKI^19,20,21^. The authors conclude that, given the inevitable use of distant donors, heart transplant centers should be committed to properly managing grafts, investing in infrastructure and transportation logistics, and evaluating the cost-effectiveness of alternative strategies that enable better cryopreservation of organs to be transplanted. A Brazilian study published by Atik et al. showed that cold ischemia time exceeding 180 minutes was associated not only with an increased need for AKS, but also with higher mortality among patients undergoing heart transplantation^26^.

There was no statistically significant difference in mortality between patients who progressed to AKI and those who did not; however, the difference was clinically relevant: those with AKI had a 20% higher mortality rate. The absence of a statistically significant difference is due to the small sample size.

It is important to highlight that most of the analyzed patients (80%) were prioritized on the heart transplant waiting list, meaning they were hospitalized and awaiting surgery, requiring invasive cardiovascular support measures (inotropic drugs and/or mechanical support), which indicates the severity of the cases studied.

The present study has some limitations, including its retrospective design, its single-center setting, and the small number of patients enrolled. Another important limitation concerns the absence of certain data in the medical records, such as plasma concentrations of calcineurin inhibitors, information on donors, and long-term follow-up data, which limited the analysis of these variables and outcomes. However, the study fulfilled its purpose by contributing to the identification of potential predictors for AKI after heart transplantation in an incipient service.

In conclusion, AKI in the postoperative period of heart transplant surgeries is frequent and is associated with preoperative factors, such as CKD; intraoperative factors, such as cold ischemia time; and postoperative factors, such as the need for VAD and their doses beyond the first postoperative day. Although this study was conducted in a recently established service in the inland region of São Paulo state, both the incidence of AKI and the risk factors associated with its development are comparable to those described in the literature^24,25^.

Further Latin American and Brazilian studies with larger samples are needed to better understand and recognize the risk factors for AKI in patients undergoing heart transplantation, with the aim of developing strategies to reduce its incidence and the resulting morbidity and mortality.

## Data Availability

Data will be available in an Excel spreadsheet if reviewers deem it necessary.

## References

[1] Brown N, Sullo N, Tyson N, Eagle-Hemming B, Lai FY, Sheikh S, Tomkova K, Joel-David L, Kumar T, Aujla H, Goodall AH, Murphy GJ, Wozniak MJ (2025). Acute kidney injury after cardiac surgery is associated with platelet activation.. J Thromb Haemost.

[2] Karkouti K, Wijeysundera DN, Yau TM, Callum JL, Cheng DC, Crowther M (2009). Acute kidney injury after cardiac surgery: focus on modifiable risk factors.. Circulation.

[3] Lagny MG, Jouret F, Koch JN, Blaffart F, Donneau AF, Albert A (2015). Incidence and outcomes of acute kidney injury after cardiac surgery using either criteria of the RIFLE classification.. BMC Nephrol.

[4] Thongprayoon C, Lertjitbanjong P, Hansrivijit P, Crisafio A, Mao MA, Watthanasuntorn K (2019). Acute kidney injury in patients undergoing cardiac transplantation: a meta-analysis.. Medicines (Basel).

[5] Nadim MK, Forni LG, Bihorac A, Hobson C, Koyner JL, Shaw A (2018). Cardiac and vascular surgery-associated acute kidney injury: The 20th International Consensus Conference of the ADQI (Acute Disease Quality Initiative) Group.. J Am Heart Assoc.

[6] Yang X, Zhu L, Pan H, Yang Y (2024). Cardiopulmonary bypass associated acute kidney injury: better understanding and better prevention.. Ren Fail.

[7] Harky A, Joshi M, Gupta S, Teoh WY, Gatta F, Snosi M (2020). Acute kidney injury associated with cardiac surgery: a comprehensive literature review.. Braz J Cardiovasc Surg.

[8] Sikma MA, Hunault CC, Kirkels JH, Verhaar MC, Kesecioglu J, de Lange DW (2018). Association of whole blood tacrolimus concentrations with kidney injury in heart transplantation patients.. Eur J Drug Metab Pharmacokinet.

[9] Ivey-Miranda JB, Flores-Umanzor E, Farrero-Torres M, Santiago E, Cepas-Guillen PL, Perez-Villa F (2018). Predictors of renal replacement therapy after heart transplantation and its impact on long-term survival.. Clin Transplant.

[10] Kidney Disease: Improving Global Outcomes (KDIGO) Acute Kidney Injury Work Group (2012). KDIGO Clinical Practice Guideline for Acute Kidney Injury.. Kidney Int Suppl.

[11] Bacal F, Marcondes-Braga FG, Rohde LEP, Xavier JL, de Souza Brito F, Moura LZ (2018). 3ª Diretriz Brasileira de Transplante Cardíaco.. Arq Bras Cardiol.

[12] Romeo FJ, Varela CF, Vulcano N, Pizarro R, Greloni G, Posatini R (2018). Acute kidney injury after cardiac transplantation: foe or common innocent bystander?. Transplant Proc.

[13] Lee JH, Yeom SY, Hwang HY, Choi JW, Cho HJ, Lee HY (2016). Twenty-yearexperience of heart transplantation: early and long-term results.. Korean J Thorac Cardiovasc Surg.

[14] Tjahjono R, Connellan M, Granger E (2016). Predictors of acute kidney injury in cardiac transplantation.. Transplant Proc.

[15] Jahangirifard A, Ahmadi ZH, Naghashzadeh F, Sharif-Kashani B, Rashid-Farokhi F, Afshar A (2018). Prophylactic fibrinogen decreases postoperative bleeding but not acute kidney injury in patients undergoing heart transplantation.. Clin Appl Thromb Hemost.

[16] De Santo LS, Romano G, Amarelli C, Maiello C, Baldascino F, Bancone C (2011). Implications of acute kidney injury after heart transplantation: what a surgeon should know.. Eur J Cardiothorac Surg..

[17] Fortrie G, Manintveld OC, Caliskan K, Bekkers JA, Betjes MG (2016). Acute Kidney Injury as a complication of cardiac transplantation: incidence, risk factors, and impact on 1-year mortality and renal function.. Transplantation.

[18] Nadkarni GN, Chauhan K, Patel A, Saha A, Poojary P, Kamat S (2017). Temporal trends of dialysis requiring acute kidney injury after orthotopic cardiac and liver transplant hospitalizations.. BMC Nephrol.

[19] Marasco SF, Esmore DS, Richardson M, Bailey M, Negri J, Rowland M (2007). Prolonged cardiac allograft ischemic time–no impact on long-term survival but at what cost?. Clin Transplant.

[20] Rylski B, Berchtold-Herz M, Olschewski M, Zeh W, Schlensak C, Siepe M (2010). Reducing the ischemic time of donor hearts will decrease morbidity and costs of cardiac transplantations.. Interact Cardiovasc Thorac Surg.

[21] Hariri G, Henocq P, Coutance G, Mansouri S, Tohme J, Guillemin J (2024). Perioperative risk factors of acute kidney injury after heart transplantation and one-year clinical outcomes: a retrospective Cohort Study.. J Cardiothorac Vasc Anesth.

[22] Hsu RK, Hsu CY (2016). The role of acute kidney injury in chronic kidney disease.. Semin Nephrol.

[23] Jocher BM, Schilling JD, Fischer I, Nakajima T, Wan F, Tanaka Y (2021). Acute kidney injury post-heart transplant: an analysis of perioperative risk factors.. Clin Transplant.

[24] Fortrie G, Manintveld OC, Caliskan K, Bekkers JA, Betjes MG (2016). Acute Kidney Injury as a complication of cardiac transplantation: incidence, risk factors, and impact on 1-year mortality and renal function.. Transplantation.

[25] Türker M, Zeyneloglu P, Sezgin A, Pirat A, Arslan G (2013). RIFLE criteria for acute kidney dysfunction following heart transplantation: incidence and risk factors.. Transplant Proc.

[26] Atik FA, Couto CF, Souza SEM, Biondi RS, Silva AHM, Vilela MF (2022). Outcomes of orthotopic heart transplantation in the setting of acute kidney injury and renal replacement therapy.. J Cardiothorac Vasc Anesth.

