# Supplementary material for: Acute kidney injury in patients undergoing cardiac transplantation: a cohort study on incidence and risk factors in the first three years of service implementation
Source: J Bras Nefrol. 2026 Mar 9;48(1):e20250135. doi: 10.1590/2175-8239-JBN-2025-0135en (PMC12970627; doi:10.1590/2175-8239-JBN-2025-0135en)
Supplement: Translation

# Translation: Injúria renal aguda em pacientes submetidos a transplante cardíaco: estudo de coorte sobre incidência e fatores de risco nos três primeiros anos de implantação do serviço

**DOI:** 10.1590/2175-8239-JBN-2025-0135pt

**Authors:** Mariana Moura Ferreira, Marcello Laneza Felicio, Flávio de Souza Brito, Leonardo Rufino Garcia, Daniela Ponce

**Affiliations:** 1 Universidade Estadual Paulista “Júlio de Mesquita Filho” (UNESP), Faculdade de Medicina de Botucatu, Curso de Medicina, Botucatu, SP, Brasil.; 2 Universidade Estadual Paulista “Júlio de Mesquita Filho” (UNESP), Faculdade de Medicina de Botucatu, Serviço de Cirurgia Cardiovascular, Botucatu, SP, Brasil.; 3 Hospital Estadual de Bauru, Serviço de Cirurgia Cardíaca, Bauru, SP, Brasil.; 4 Universidade Estadual Paulista “Júlio de Mesquita Filho” (UNESP), Faculdade de Medicina de Botucatu, Programa de Transplante Cardíaco, Botucatu, SP, Brasil.; 5 Centro de Pesquisa Clínica Indacor, Indaiatuba, SP, Brasil.; 6 Faculdade de Medicina UniMAX, Curso de Medicina, Indaiatuba, SP, Brasil.; 7 Universidade Estadual Paulista “Júlio de Mesquita Filho” (UNESP), Faculdade de Medicina de Botucatu, Unidade de Diálise do Hospital das Clínicas, Botucatu, SP, Brasil.

**Keywords:** Injúria Renal Aguda, Transplante de Coração, Ponte Cardiopulmonar; Circulação, Extracorpórea, Mortalidade, Fatores de Risco

## Abstract

**Introdução:**

A injúria renal aguda (IRA) é uma complicação frequente no período pós-operatório do transplante cardíaco, associando-se a um prognóstico desfavorável dos pacientes.

**Objetivo:**

Analisar a incidência de IRA em pacientes submetidos a transplante cardíaco e identificar fatores de risco pré-operatórios, intraoperatórios e pósoperatórios associados ao seu desenvolvimento.

**Métodos:**

Estudo de coorte retrospectivo e unicêntrico, que avaliou pacientes submetidos a transplante cardíaco nos três primeiros anos de implantação do serviço em um hospital terciário do Estado de São Paulo, no período de janeiro de 2020 a janeiro de 2023. Foram excluídos pacientes com doença renal crônica (DRC) estágios 4 e 5, bem como transplantados renais.

**Resultados:**

A incidência de IRA foi de 48%. A análise de regressão logística evidenciou associação entre IRA e os seguintes fatores: DRC prévia (OR = 3,155; IC = 1,343–6,340; p = 0,031), tempo de isquemia fria (OR = 1,956; IC = 1,126–3,053; p = 0,042) e maiores doses de noradrenalina no primeiro pós-operatório (OR = 5,211; IC = 2,696–8,987; p = 0,028). Não houve diferença na mortalidade entre os pacientes que evoluíram ou não com IRA (58,3% vs. 38,5%; p = 0,09).

**Conclusão:**

Houve elevada incidência de IRA (48%) nesta população, e os principais fatores de risco para seu desenvolvimento foram DRC prévia, tempo de isquemia fria e maiores doses de noradrenalina no primeiro pós-operatório.

## Introdução

A injúria renal aguda (IRA) constitui uma complicação frequente em pacientes submetidos à cirurgia de transplante cardíaco, associando-se a uma maior morbidade e mortalidade, além de prolongar a internação e elevar os custos hospitalares^1,2,3,4^. De acordo com uma revisão sistemática publicada por Thongprayoon e cols. em 2019, a incidência de IRA após transplante cardíaco foi de 62%^4^. Portanto, sua prevenção e o conhecimento de sua fisiopatologia são tópicos de interesse.

A fisiopatologia da IRA não é única e associase à sua própria etiologia. A lesão pode ter origem em hipoperfusão renal, exposição a drogas nefrotóxicas, sepse e imunossupressão, entre outras causas^1,2 3,4^.

Dentre os fatores de risco identificados, encontram-se os pré-operatórios, incluindo a doença renal crônica (DRC), o diabetes mellitus (DM) e a idade avançada^5,6,7^. Os fatores de risco perioperatórios e pós-operatórios (PO) incluem o aumento do tempo de circulação extracorpórea (CEC) e de cirurgia, o prolongamento do tempo de isquemia fria (TIF), a concentração supraterapêutica de inibidores de calcineurina, o sangramento PO, a necessidade de dispositivo de assistência ventricular direita, a duração da ventilação mecânica (VM) e a necessidade de oxigenação por membrana extracorpórea (ECMO) após o transplante cardíaco^8,9,10^.

Apesar dos enormes avanços ocorridos na medicina, especificamente nas áreas de transplante cardíaco e nefrologia, ainda há escassez de dados sobre a incidência e os fatores de risco associados à IRA em pacientes submetidos a transplante cardíaco, principalmente em países em desenvolvimento.

A identificação desses fatores de risco permite o direcionamento de condutas voltadas à sua minimização. Dessa forma, este estudo tem por objetivo identificar os fatores de risco que possam aumentar as chances de o paciente transplantado cardíaco desenvolver IRA na primeira semana de PO, sejam eles prévios, presentes no ato cirúrgico, ou posteriores à cirurgia.

## Métodos

Foi realizado um estudo retrospectivo observacional do tipo coorte, envolvendo pacientes adultos submetidos a transplante cardíaco no Hospital das Clínicas da Faculdade de Medicina de Botucatu (HCFMB), no período de janeiro de 2020 a janeiro de 2023.

Foram excluídos do estudo os pacientes com DRC estágios 4 e 5 (*clearance* de creatinina inferior a 30 ml/min), os transplantados renais e aqueles que apresentaram IRA previamente ao transplante cardíaco.

O estudo foi analisado e aprovado pelo Comitê de Ética em Pesquisa da Faculdade de Medicina de Botucatu (FMB), sob o parecer n° 5.977.493, sendo iniciado somente após a sua aprovação.

Foram coletados dados pré-operatórios como idade, peso, altura (com cálculo do índice de massa corporal – IMC), presença de comorbidades como diabetes, hipertensão e obesidade, creatinina basal, índices prognósticos, etiologia da insuficiência cardíaca e dados do ecocardiograma (fração de ejeção [FE], espessura do septo e da parede posterior, hipertensão pulmonar). Também foram obtidos dados intraoperatórios, como duração da cirurgia, tempo de isquemia fria, duração da CEC e intercorrências intraoperatórias, incluindo instabilidade hemodinâmica, necessidade de drogas vasoativas (DVA) e de transfusão sanguínea. Além disso, foram avaliadas diariamente as seguintes variáveis clínicas e laboratoriais, tanto no préoperatório quanto nos três primeiros dias do PO: diurese, necessidade de ventilação mecânica e fração de oxigênio ofertada (FiO2), uso de drogas vasoativas, temperatura, hemoglobina, albumina, proteína C reativa e contagem de leucócitos.

A definição e a classificação da IRA utilizadas neste estudo foram as adotadas pela *Kidney Disease Improving Global Outcomes* (KDIGO) de 2012, baseando-se tanto na elevação da creatinina (≥0,3 mg/dl em 48 horas ou ≥1,5 em 7 dias) quanto na diminuição do débito urinário (<0,5 ml/kg/h por mais de 6 horas)^11^.

A prescrição dos imunossupressores seguiu um protocolo institucional baseado nas Diretrizes Brasileiras de Transplante Cardíaco de 2018^12^ e consistiu em terapia tripla (prednisona, micofenolato mofetil e tacrolimus). Quando a doença de base foi chagásica, o micofenolato foi substituído por azatioprina.

A partir do protocolo de estudo, os dados foram digitados em planilha eletrônica, verificando-se possíveis erros de digitação, e sua análise foi realizada com o auxílio do programa IBM SPSS for Windows,versão 17.0. Inicialmente, foi feita uma análise descritiva dos pacientes acompanhados no período, sendo calculadas medidas de tendência central e de dispersão para as variáveis contínuas, e de frequência para as variáveis categóricas. Em seguida, estabeleceuse como variável dependente, a ocorrência de IRA.

Para a análise das variáveis categóricas, foi utilizado o teste do Qui-Quadrado; para a comparação das variáveis contínuas e paramétricas, aplicouse o teste *t*; e, para as não paramétricas, o teste de *Mann-Whitney*. Foi realizada análise multivariada por regressão logística, incluindo-se no modelo todas as variáveis que apresentaram associação com o desfecho (p ≤ 0,05) nas análises univariadas, após a exclusão daquelas consideradas colineares, identificadas pelo teste de *Kolmogorov-Smirnov*. O método de *backward selection* foi aplicado de forma seriada para remover as variáveis não significantes. As variáveis que, quando retiradas, provocavam alteração no parâmetro superior a 10% foram reintroduzidas no modelo para controle de possíveis fatores de confusão. Em todos os testes realizados, adotou-se nível de significância de 5%.

## Resultados

No período do estudo, 58 pacientes foram submetidos à cirurgia de transplante cardíaco no Hospital das Clínicas de Botucatu. Destes, oito foram excluídos por apresentarem IRA no pré-operatório ou por serem portadores de DRC estágio 4, conforme

mostra a Figura 1. A maioria dos pacientes era do sexo masculino (76%) e da raça branca (84%), com idade média de 48 ± 12 anos. As principais etiologias da insuficiência cardíaca foram a isquêmica (32%) e a chagásica (28%). As comorbidades mais frequentes foram hipertensão arterial (32%), diabetes mellitus tipo 2 (16%) e DRC (18%). A creatinina basal dos pacientes foi de 0,89 ± 0,32 mg/dl, a FE de 23,9 ± 10%, a espessura do ventrículo esquerdo (VE) de 8,9 ± 1,9 e a pressão na artéria pulmonar foi de 49,5 ± 15,5 mmHg. A grande maioria dos pacientes apresentou FE reduzida (90%) e hipertensão pulmonar (85%), conforme mostra a Tabela 1. As concentrações de hemoglobina e albumina no préoperatório foram de 11,96 ± 2,13 e 3,70 ± 0,84 d/dl, respectivamente. A grande maioria dos pacientes estava em uso de inotrópico positivo (80%), e 12 estavam em uso de balão intra-aórtico (BIA) (24%) no pré-operatório.

**Figura 1.** Fluxograma de seleção dos pacientes.

Abreviaturas – DRC: doença renal crônica; IRA: injúria renal aguda.

**Tabela 1:** Características clínicas e laboratoriais pré-operatórias dos pacientes de acordo com o desenvolvimento ou não de ira durante a internação

Variáveis	Geral (N = 50)	IRA (N = 24)	Não IRA	(N = 26) Valor P
Sexo masculino (%)	38 (76)	19 (79,2)	19 (73,1)	0,614
Idade (anos)	48 ± 12	44 ± 10	51 ± 12	0,02
Raça (%)				0,364
Branca	42 (84%)	22 (91,7%)	20 (76,9%)	
Preta	4 (8%)	1 (4,2%)	3 (11,5%)	
Comorbidades (%)				
Obesidade	4 (8)	2 (8,3)	2 (7,6)	0,709
DRC	9 (18%)	8 (33,3%)	1 (3,8%)	0,007
DM	8 (16%)	4 (16,7%)	4 (15,4%)	0,902
HAS	16 (32%)	8 (33,3%)	8 (30,8%)	0,846
Tireoidopatia	7 (14%)	5 (20,8%)	2 (7,7%)	0,274
Ex-tabagismo	13 (26%)	5 (20,8%)	8 (30,8%)	0,422
Ex-etilismo	7 (14%)	3 (12,5%)	4 (15,4%)	0,587
Outras (%)^*^	19 (38%)	10 (41,7%)	9 (34,6%)	0,575
Tipo de IC (%)				0,605
Isquêmica	16 (32%)	8 (30%)	8 (30,1%)	
Valvar	4 (8%)	2 (8,3%)	2 (7,7%)	
Chagásica	14 (28%)	9 (37,5%)	5 (19,2%)	
Outras^**^	16 (32%)	6 (25%)	8 (29,7%)	
Dados do ecocardiograma				
FE (%)	23,9 ± 10	26,1 ± 11,2	21,4 ± 8,4	0,03
Espessura do VE (mm)	8,9 ± 1,9	9,5 ± 2,1	8,5 ± 1,5	0,249
Pressão na artéria pulmonar (mmHg)	49,5 ± 15,5	51,5 ± 16,5	48,8 ± 13,9	0,263
Hemoglobina pré-operatório (g/dL)	11,96 ± 2,13	11,64 ± 2,31	12,24 ± 1,95	0,336
Albumina pré-operatório (g/dL)	3,70 ± 0,84	3,43 ± 0,85	3,90 ± 0,80	0,086
PCR pré-operatório (mg/dL)	3,93 ± 5,82	4,97 ± 7,12	2,89 ± 4,04	0,251
Leucócitos pré-operatório (n°/mm^3^)	8,51 ± 3,46	8,86 ± 3,94	8,20 ± 3,03	0,514
Uso de inotrópicos (%)	40 (80)	20 (83)	20 (76,9)	0,621
Uso de BIA (%)	12 (24)	9 (37,5)	3 (11,4)	0,108

Abreviaturas – BIA, balão intra-aórtico; DM, diabetes mellitus; DRC, doença renal crônica; DVA, droga vasoativa; FE, fração de ejeção; HAS, hipertensão arterial sistêmica; IC, insuficiência cardíaca, PCR, proteína C reativa; VE, ventrículo esquerdo.

Notas – ^*^Doença arterial crônica, Síndrome de Sjogren, transtorno depressivo, uso de drogas ilícitas, neuralgia do trigêmeo, etc.

^**^Miocardite viral, miocardiopatia dilatada a/e, isquêmica+valvar, isquêmica+chagásica.

A incidência de IRA foi de 48%. O tempo médio para o desenvolvimento da IRA foi de 2,79 ± 1,06 dias, variando de 1 a 5 dias. Houve predomínio de IRA KDIGO 3 (10 pacientes – 41,7%), seguida de IRA KDIGO 2 (7 pacientes – 29,1%) e de DRC com IRA sobreposta (6 pacientes – 25%). A indicação de suporte renal artificial (SRA) ocorreu em 19 dos pacientes (38%). A modalidade de SRA predominante foi a hemodiálise prolongada (9 pacientes – 47,4%), seguida de terapia venovenosa contínua (6 pacientes – 31,6%), de hemodiálise intermitente convencional (2 pacientes – 10,5%) e de diálise peritoneal (2 pacientes – 10,5%).

Os fatores pré-operatórios identificados como associados à IRA foram idade (44 ± 10 vs. 51 ± 12 anos; p = 0,02), FE (26,3 ± 11,2 vs. 21,4 ± 8,6; p = 0,03) e presença de DRC (3,8 vs. 33%; p = 0,007). Não houve diferença entre os pacientes com e sem IRA quanto ao sexo, comorbidades, pressão na artéria pulmonar, uso de inotrópicos ou dispositivos no pré-operatório.

Durante o período intraoperatório, a grande maioria dos pacientes necessitou de drogas vasoativas (96%) e de transfusão de hemoconcentrados (96%). O tempo total de CEC foi de 264,9 ± 59 min, o tempo de isquemia fria de 169,97 ± 75,49 min e o de isquemia quente foi de 117,71 ± 41,31 min. Os grupos com e sem IRA foram semelhantes quanto à necessidade e ao tempo de CEC, ao uso de drogas vasoativas, à necessidade e à quantidade de hemotransfusão e ao tempo de isquemia quente. Contudo, diferiram quanto ao tempo de isquemia fria, que foi maior nos pacientes que evoluíram com IRA (196,83 ± 66,10 min vs. 141,53 ± 76,22; p = 0,028), conforme mostra a Tabela 2.

**Tabela 2:** Análise de características intraoperatórias dos pacientes que evoluíram para ira na internação

Variáveis	Média Geral (N = 50)	IRA (N = 24)	Não IRA (N = 26)	Valor P
Uso de CEC (%)	47 (94%)	24 (100%)	23 (88,5%)	0,229
Uso de DVA no intraoperatório (%)				
Noradrenalina	34 (68%)	18 (75%)	16 (61,5%)	0,440
Dobutamina	48 (96%)	24 (100%)	24 (92,3%)	0,382
Milrinone	40 (80%)	20 (83,3%)	20 (76,9%)	0,597
Adrenalina	26 (52%)	14 (58,3%)	12 (46,2%)	0,480
Intercorrências cirúrgicas (%)				0,620
Sangramento importante	4 (8%)	3 (12,5%)	1 (3,8%)	
Choque vasoplégico	2 (4%)	1 (4,2%)	1 (3,8%)	
Outras	6 (12%)	4 (16,7%)	2 (7,7%)	
Tempo total de CEC (minutos)	264,89 ± 59,97	274,13 ± 68,05	254,82 ± 49,32	0,280
Tempo de isquemia fria (minutos)	169,97 ± 75,49	196,83 ± 66,10	141,53 ± 76,22	0,028
Tempo de isquemia quente (minutos)	117,71 ± 41,31	125,12 ± 26,50	110,29 ± 51,97	0,303
Uso de hemoderivados – concentrado de hemácias (unidade)	4,09 ± 2,79	4,43 ± 2,42	3,74 ± 3,13	0,405
Uso de hemoderivados – plasma fresco congelado (unidade)	2,59 ± 1,99	2,61 ± 1,87	2,57 ± 2,15	0,942
Uso de hemoderivados – crioprecipitado (unidade)	2,87 ± 3,27	2,74 ± 3,29	3,00 ± 3,31	0,790
Uso de hemoderivados – plaquetaférese (unidade)	0,99 ± 0,62	1,09 ± 0,57	0,89 ± 0,67	0,296

Abreviaturas – DVA, droga vasoativa; CEC, circulação extracorpórea.

Os fatores associados à IRA no 1º dia de PO foram necessidade de DVA (95,8 vs. 65,4%; p = 0,034), dose de noradrenalina (0,57 ± 0,23 vs. 0,18 ± 0,06 mcg/kg/min; p = 0,041), valores mais elevados de PCR (16,78 ± 9,23 vs. 10,77 ± 6,72 mg/dl; p = 0,026) e maior necessidade de ventilação mecânica (50 vs. 15,4%; p = 0,014). Não houve diferença entre os pacientes com e sem IRA quanto à FiO2, temperatura máxima, valores de hemoglobina, albumina e leucócitos, conforme mostra a Tabela 3.

**Tabela 3:** Variáveis analisadas no 1° e 3° dia pós-operatório dos pacientes quanto ao desenvolvimento de ira na internação

Variáveis	Média Geral (N = 50)	IRA (N = 24)	Não IRA (N = 26)	Valor P
FiO2 1° PO (%)	49,68 ± 21,63	54,16 ± 23,33	36,25 ± 14,78	0,158
Uso de DVA (%)	40 (80)	23 (95,8)	17 (65,4)	0,034
DVA 1° PO – noradrenalina (mcg/kg/min)	0,36 ± 0,62	0,57 ± 0,53	0,18 ± 0,66	0,041
DVA 1° PO – dobutamina (mcg/kg/min)	9,47 ± 3,22	9,65 ± 3,71	9,33 ± 2,81	0,749
DVA 1° PO – milrinone (mcg/kg/min)	0,49 ± 0,29	0,52 ± 0,33	0,46 ± 0,26	0,564
DVA 1° PO – adrenalina (mcg/kg/min)	0,24 ± 0,12	0,42 ± 0,25	0,08 ± 0,06	0,008
Temperatura máxima 1° PO (°C)	37,44 ± 0,92	37,67 ± 1,06	37,24 ± 0,73	0,132
Hemoglobina 1° PO (g/dL)	9,12 ± 1,23	9,52 ± 1,09	8,94 ± 1,30	0,127
Albumina 1° PO (g/dL)	2,68 ± 0,43	2,67 ± 0,48	2,70 ± 0,38	0,826
PCR 1° PO (mg/dL)	13,30 ± 8,32	16,78 ± 9,23	10,77 ± 6,72	0,026
Leucócitos 1° PO (n°/mm^3^)	16,72 ± 7,39	18,05 ± 8,76	15,56 ± 5,92	0,277
Ventilação mecânica no 1° PO (%)	16 (32%)	12 (50%)	4 (15,4%)	0,014
FiO2 3° PO (%)	37,77 ± 11,48	38,75 ± 11,87	30,00	0,510
Uso de DVA (%)	32 (64)	18 (75)	14 (53,8)	0,314
DVA 3° PO – noradrenalina (mcg/kg/min)	0,24 ± 0,51	0,56 ± 0,66	0,00	<0,001
DVA 3° PO – dobutamina (mcg/kg/min)	8,95 ± 3,29	10,35 ± 3,47	7,86 ± 2,76	0,015
DVA 3° PO – milrinone (mcg/kg/min)	0,50 ± 0,29	0,56 ± 0,28	0,46 ± 0,30	0,300
DVA 3° PO – adrenalina (mcg/kg/min)	0,07 ± 0,21	0,15 ± 0,30	0,01 ± 0,04	0,036
Temperatura máxima 3° PO (°C)	36,60 ± 0,57	36,46 ± 0,59	36,71 ± 0,55	0,179
Hemoglobina 3° PO (g/dL)	8,77 ± 1,23	9,07 ± 1,68	8,53 ± 0,67	0,170
Albumina 3° PO (g/dL)	2,73 ± 0,37	2,70 ± 0,39	2,77 ± 0,36	0,568
PCR 3° PO (mg/dL)	9,83 ± 933	12,76 ± 11,98	7,92 ± 6,73	0,119
Leucócitos 3° PO (n°/mm^3^)	15,31 ± 7,28	17,66 ± 8,48	13,47 ± 5,71	0,067
Ventilação mecânica no 3° PO (%)	8 (16%)	7 (29,2%)	1 (3,8%)	0,011
Uso de drogas nefrotóxicas nos 3 primeiros PO (aminoglicosídeos e/ou vancomicina)	14 (28%)	8 (33,3%)	6 (23,1%)	0,317

Abreviaturas – DVA, droga vasoativa; FiO2, fração inspirada de oxigênio; PAD, pressão arterial diastólica; PAS, pressão arterial sistólica; PCR, proteína C reativa; PO, pós-operatório.

No 3° dia de PO, a necessidade de ventilação mecânica (29,2% vs. 3,8%; p = 0,011) e maiores doses de noradrenalina permaneceram associadas à incidência de IRA (0,56 ± 0,66 vs. 0; p < 0,001). Os demais parâmetros, como FiO2, outras drogas vasoativas utilizadas, temperatura máxima, hemoglobina, albumina, proteína C reativa, leucometria e uso de drogas nefrotóxicas (como aminoglicosídeos e vancomicina), não se associaram à IRA, conforme observado na Tabela 3. Todos os pacientes incluídos no estudo receberam inibidores de calcineurina no esquema de imunossupressão.

Por fim, realizou-se análise multivariada por meio da construção de um modelo de regressão logística utilizando o método *backward stepwise*. Foram incluídas no modelo as variáveis FE, DRC, TIF e dose de noradrenalina no 1º PO, todas consideradas clinicamente relevantes e com p < 0,05 na análise univariada. Foram excluídas as variáveis idade, uso de DVA, PCR e necessidade de VM por terem sido identificadas como colineares com a FE e a dose de noradrenalina no 1º PO.

Ao realizar a análise de regressão logística, apresentada na Tabela 4, observou-se que a DRC, o tempo de isquemia fria e a dose de noradrenalina no 1º PO associaram-se independentemente à ocorrência de IRA, conforme mostra a tabela.

**Tabela 4:** Análise de regressão logística para ira

Variável	OR	IC 95%	Valor P
DRC	3,155	1,343–6,340	0,031
Tempo de isquemia fria (min)	1,956	1,126–3,053	0,042
FE (%)	1,423	0,962–2,784	0,145
Dose de noradrenalina no 1º PO (0,1 mcg/kg/min)	5,211	2,696–8,987	0,041

Abreviaturas – DRC, doença renal crônica; FE, fração de ejeção; PO, pós-operatório.

A mortalidade geral dos pacientes submetidos a transplante cardíaco foi de 42%, não havendo diferença entre aqueles com e sem IRA (58,3 vs. 38,5%; p = 0,09).

## Discussão

Nossos resultados demonstram que a incidência de IRA no pós-operatório de pacientes submetidos a transplante cardíaco é elevada (48%) e ocorre, em média, 2,79 ± 1,06 dias após a cirurgia, com predomínio de IRA KDIGO 3 (41,7%). A presença de DRC, o maior tempo de isquemia fria e a maior dose de noradrenalina no primeiro pós-operatório estão associados à ocorrência de IRA em pacientes submetidos ao transplante cardíaco.

De acordo com revisão sistemática publicada por Thongprayoon e cols. em 2019, a incidência de IRA no PO do transplante cardíaco é elevada, chegando a 62,8%. Portanto, sua prevenção e o reconhecimento dos fatores de risco são necessários, visto que a IRA associa-se a maiores taxas de mortalidade. No entanto, ainda são escassos os estudos realizados em populações latino-americanas e em países em desenvolvimento.

Estudos de coorte anteriores identificaram diversos fatores de risco perioperatórios, intraoperatórios e pós-operatórios para o desenvolvimento de IRA em transplantados cardíacos, tais como maior idade, presença de comorbidades (incluindo DRC, diabetes, hipertensão e tabagismo), tempo prolongado de isquemia e de CEC, necessidade de ECMO, concentrações supraterapêuricas dos inibidores de calcineurina, hipertensão pulmonar, duração da necessidade de ventilação mecânica e uso de noradrenalina, além da politransfusão^3,4,5,6,7,8,13,14,15,16,17^.

Em consonância com a literatura atual, nossos resultados mostram que idade^16,17,18^, presença de DRC^14,15,16,17^, menor FE, maior tempo de isquemia fria^17^, necessidade de DVA e de ventilação mecânica no primeiro e no terceiro dia do pós-operatório^15,16,17^, além de maiores níveis de PCR, associam-se ao desenvolvimento da IRA. No entanto, dentre todas essas variáveis, apenas a presença de DRC, o tempo de isquemia fria e as maiores doses de noradrenalina permaneceram significativas em nossa regressão logística multivariada.

A DRC é um fator importante a ser considerado, pois pacientes que apresentam essa condição já possuem função renal comprometida, o que aumenta a vulnerabilidade renal a fatores de estresse durante e após o procedimento cirúrgico. A redução da reserva renal torna sua resposta insuficiente às variações de pressão, ao eventual uso de drogas nefrotóxicas e a outros possíveis insultos renais. Por esse motivo, a DRC é considerada um fator de risco para a ocorrência de IRA^14,15,16,17^. Ademais, a DRC pode estar presente em pacientes com cardiopatias mais graves, ou seja, naqueles com síndrome cardiorrenal tipo 2, funcionando como um marcador indireto da gravidade da própria insuficiência cardíaca. No presente estudo, houve colinearidade entre creatinina basal, FE e idade, o que corrobora com essa possível associação. A senilidade renal torna as respostas renais e sua autorregulação mais lentas, aumentando a susceptibilidade à IRA. Além disso, o envelhecimento natural dos rins pode reduzir sua capacidade de recuperação após eventos de estresse, como a cirurgia de transplante cardíaco^13,21,22,23,24,25^.

Estudos prévios também identificaram o uso de drogas vasoativas como um preditor de IRA. Tais fármacos são frequentemente necessários para manter a pressão arterial média acima de 65 mmHg durante e após a cirurgia^15,16,17^. No entanto, quando seu uso se faz necessário após o PO imediato, isso pode refletir complicações associadas ao procedimento cirúrgico, como sangramentos intensos, ou estar associado a quadros infecciosos. No presente estudo, doses mais elevadas de noradrenalina no primeiro pós operatório associaram-se ao desenvolvimento de IRA, o que provavelmente reflete complicações infecciosas adquiridas, uma vez que houve colinearidade entre dose de vasopressores, concentrações plasmáticas de PCR e necessidade de ventilação mecânica.

Por fim, poucos estudos evidenciaram que o tempo de isquemia fria – ou seja, o período em que o coração transplantado permanece sem circulação sanguínea e oxigênio durante o procedimento – impacta no risco de IRA^17^. Quanto maior esse tempo, maior o risco de danos ao coração transplantado e também a outros órgãos, como os rins.

O tempo de isquemia do enxerto e a liberação de troponina são preditores conhecidos de baixo desempenho inicial do enxerto^17^. A escassez de oferta de enxertos cardíacos implica a necessidade de avaliação e utilização de doadores marginais e geograficamente distantes. O centro transplantador do presente estudo está localizado em um país de dimensões continentais, a aproximadamente 250 km da capital paulista. A isquemia prolongada resulta em maiores danos sistêmicos, decorrentes do estresse oxidativo, da inflamação e da própria isquemia. Dois estudos prévios evidenciaram o impacto desse fenômeno na morbidade pós-operatória precoce, incluindo falência primária do enxerto, necessidade de ECMO e ocorrência de IRA^19,20,21^. Os autores concluem que, sendo inevitável o uso de doadores distantes, os centros transplantadores cardíacos devem estar comprometidos em gerenciar adequadamente os enxertos, investir em infraestrutura e logística para o transporte, além de avaliar a relação custo-efetividade de estratégias alternativas que possibilitem melhor criopreservação dos órgãos a serem transplantados. Um estudo brasileiro, publicado por Atik e cols., mostrou que o tempo de isquemia fria superior a 180 minutos associou-se não apenas a uma maior necessidade de SRA, mas também à maior mortalidade entre os pacientes submetidos ao transplante cardíaco^26^.

Não houve diferença estatisticamente significativa na mortalidade dos pacientes que evoluíram ou não com IRA; entretanto, a diferença foi clinicamente relevante: aqueles com IRA apresentaram mortalidade 20% maior. A ausência de uma diferença estatisticamente significativa deve-se ao reduzido tamanho amostral.

EÉ importante ressaltar que a maioria dos pacientes analisados (80%) encontrava-se priorizada na lista de transplante cardíaco, ou seja, hospitalizada e aguardando o momento cirúrgico, com necessidade de medidas invasivas de suporte cardiovascular (drogas inotrópicas e/ou suporte mecânico), o que denota a gravidade dos casos estudados.

O presente estudo apresenta algumas limitações, como o desenho retrospectivo, a realização em um único centro e o pequeno número de pacientes incluídos. Outra importante limitação diz respeito à ausência de alguns dados nos prontuários, como a concentração plasmática dos inibidores de calcineurina, informações sobre os doadores e dados de seguimento em longo prazo, os quais limitaram a análise dessas variáveis e desfechos. No entanto, o estudo cumpriu sua proposta ao contribuir para a identificação de possíveis fatores preditores de IRA após o procedimento de transplante cardíaco em um serviço incipiente.

Como conclusão, a IRA no PO de cirurgias de transplante cardíaco é frequente e associa-se a fatores pré-operatórios, como DRC; intraoperatórios, como o tempo de isquemia fria; e PO, como a necessidade de DVA e suas doses além do primeiro dia de PO. Embora se trate de um serviço recentemente implantado no interior do estado de São Paulo, tanto a incidência de IRA quanto os fatores de risco para seu desenvolvimento assemelham-se aos descritos na literatura^24,25^.

Mais estudos latino-americanos e brasileiros, com amostras maiores, são necessários para uma melhor compreensão e o reconhecimento dos fatores de risco para IRA em pacientes submetidos ao transplante cardíaco, com o objetivo de desenvolver estratégias que reduzam sua incidência e consequente morbimortalidade.

## Data Availability

Os dados serão disponíveis em planilha de Excel caso os revisores julguem necessário.
